# A brief history of the German Society for Experimental and Clinical Pharmacology and Toxicology—an overview of available sources

**DOI:** 10.1007/s00210-026-05197-w

**Published:** 2026-03-28

**Authors:** Jessica Marie Steinert, Roland Seifert

**Affiliations:** Institute of Pharmacology, Hannover Medical School, Carl-Neuberg-Str. 1, 30625 Hannover, Germany

**Keywords:** DGPT history, DGPT archive, Medical history research

## Abstract

The history of the German Society for Experimental and Clinical Pharmacology and Toxicology (DGPT), which has played an important role in pharmacology in Germany and Europe for more than 100 years, is examined in this article. Many studies have addressed parts of this history, but they usually focus on short periods or specific topics. This article tries to bring together and explain the main sources on the DGPT and the development of pharmacology in German-speaking countries. Important references include early works by Philippu and Seifert, chronological surveys by Lindner, and the multi-volume histories by Philippu. Personal and institutional sources, such as Wolfgang Heubner’s diaries and the DGPT Archive curated by Erich Muscholl, are also discussed. Using the sources of the archive, several articles about the DGPT’s history have been published. The society’s history during the Nazi era, the division of Germany after 1945, the reunification of German pharmacology, and the development of its journals and organizational structure are reviewed. Rather than providing a full historical account, this article offers a clear guide to the most relevant sources and lays the groundwork for future research on the DGPT.

## Introduction

The German Society for Experimental and Clinical Pharmacology and Toxicology (DGPT) is one of the key scientific institutions in the development of modern pharmacology in Germany and Europe. For more than 100 years, it has shaped and influenced the scientific landscape in a lasting way.

The importance of the society is reflected in the numerous publications that deal with the history of German pharmacology in general and of the DGPT and its precursors in particular. However, these studies usually focus on limited time periods or specific topics. So far, there is no comprehensive work that systematically reviews the available sources over the whole timeframe.

Even figures such as Wolfgang Heubner, who shaped the history of pharmacology, recognized the value of historical work. As noted in Lindner’s preface, he states about Lindner: “It shows a remarkable level of intellect when a young researcher of the natural sciences, like the author of this work, becomes so interested in historical and humanities-related questions that they choose to devote their own efforts to exploring them” (Lindner 1957). Even at that time, the importance of studying history was understood, which of course is still relevant to this day. The aim of this article is to provide an overview of the material on the history of the DGPT and on pharmacology in the German-speaking world. In doing so, it tries to offer insight into the dynamic of the history of the society and, above all, to lay the groundwork for further research.

This article does not offer a full historical account. Instead, it provides an accessible compilation of the most relevant sources that serves as a point of orientation for those interested in the history of the DGPT and is intended to encourage further research on the topic.

## Sources in context

### Overview literature of the history and development of pharmacology

To understand the history of the DGPT, it is essential to consider the early development of pharmacology as a scientific discipline. Early pioneers such as Buchheim and Schmiedeberg played an important role in establishing pharmacology as an independent academic field. Their foundational work has been well documented in two studies by Philippu and Seifert (Philippu and Seifert 2023a, 2023b) which both show the origins and international spread of pharmacology by telling the history of the important pharmacological institutes of Tartu and Strasbourg.

The first part of their two-part publication “History of pharmacology: 1—the Department of Pharmacology of the University of Tartu (Dorpat): genealogy and biographies” (Philippu and Seifert 2023a) explores the establishment of pharmacology in Tartu (Dorpat), Estonia, by Naunyn, Buchheim, and Schmiedeberg, highlighting how scientific vision and academic freedom rather than wealth or institutional prestige fueled its growth. They provide brief biographies of the institute’s professors, tracing their lives and work up to the present day.

The second part “History of pharmacology:2—The Institute of Pharmacology of the University of Strasbourg: genealogy and biographies” (Philippu and Seifert 2023b) focuses on the Institute of Pharmacology in Strasbourg (1872–1918), where Oswald Schmiedeberg trained an entire generation of pharmacologists and profoundly shaped academic pharmacology. The institute’s evolution also reflects the political developments between Germany and France, including the Nazi period.

**Fig. 1:**
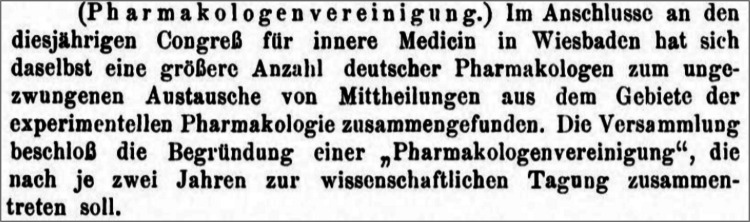
Announcement of the founding of the “Pharmakologenvereinigung” 1900 in the Wiener Medizinische Presse, Nr. 22. p. 1027 (Wiener Medizinische Presse 1900, 22:1027)

Another key reference in early literature is Jürgen Lindner’s *Zeittafeln zur Geschichte der Pharmakologischen Institute des deutschen Sprachgebietes* (Lindner 1957) which was completed before his death and published after in 1957. This work includes one of the few documented mentions of a pharmacologists’ association (“Pharmakologenvereinigung”) founded in 1900, which predates the Deutsche Pharmakologische Gesellschaft (DPhG), the DGPT’s predecessor. Lindner’s book presents chronological tables of pharmacological institutes and industrial laboratories in German-speaking regions, documenting their development, leadership, and short biographic tables of notable academic figures. It is cited by many later works. A second edition, revised by Lüllmann, appeared in 1996 (Lüllmann 1996). Another mention of the “Pharmakologenvereinigung” appears in the Wiener Medizinische Presse, which reported on the association shortly after its establishment in 1900 (Fig. 1).

**Fig. 2:**
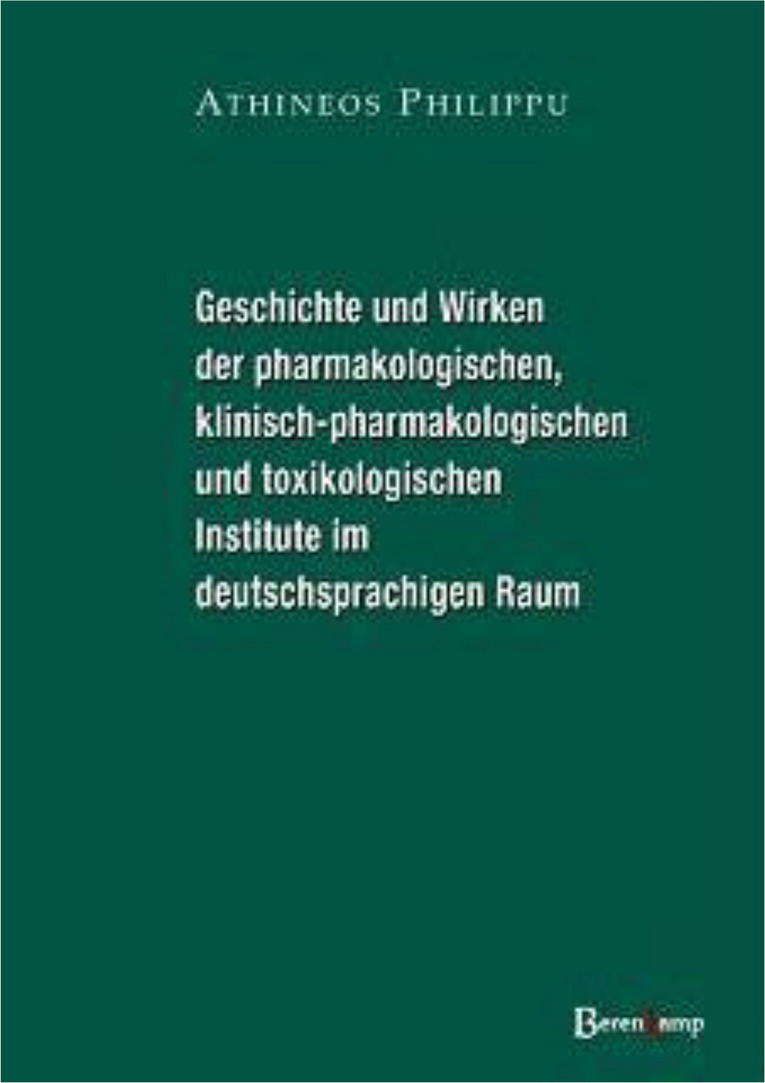
Cover of volume 1 of Philippus six volume series: *Geschichte und Wirken der pharmakologischen und klinisch-pharmakologischen und toxikologischen Institute im deutschsprachigen Raum* (Philippu 2004)
(II) Volume two mainly presents historical photographs and illustrations of the pharmacological institutes in German-speaking countries and influential figures of the field. It complements the text volumes by visually showing the history of the institutes, their facilities, and key figures (Philippu 2007).
(III) Volume three supplements the texts of Volume I and the images of Volume II with updates, reflecting recent developments at the institutes and by adding institutions that were previously not included. It also documents the histories of former German universities (Philippu 2011).
(IV) Volume four, comprising 876 pages, presents the autobiographies of 62 leading toxicologists and clinical pharmacologists. Although the prologue describes this volume as the final part of the work, two additional volumes were published later (Philippu 2014, p. 7) (Philippu 2014).
(V) Volume five continues and expands the biographical section of the series. It adds 11 important autobiographies and includes 35 biographies written by colleagues and contemporaries of the individuals portrayed. These contributions offer more personal insights into the lives, careers, and characters of the featured scientists (Philippu 2017).
(VI) Volume six contains a further 15 autobiographies and 13 biographies, following the approach of Volume V. It also includes the tables of contents of all previous volumes, which makes it easier to gain an overview of the entire series (Philippu 2021).

Athineos Philippu’s six-volume series *Geschichte und Wirken der pharmakologischen, klinisch-pharmakologischen und toxikologischen Institute im deutschsprachigen Raum* (Philippu 2004, 2007, 2011, 2014, 2017, 2021) can be viewed as a more recent and extensive continuation of Lindner’s effort. The series provides a comprehensive overview of both university and non-university pharmacological institutions, includes (auto)biographies of influential pharmacologists from the twentieth century to the present day, and features one volume designed as an illustrated book (“Bildband”). The following provides a brief overview of each volume:

(I) In the first volume alone (Fig. 2), the work traces the development of pharmacology, clinical pharmacology, and toxicology institutes from the nineteenth century to the present day. Across approximately 1000 pages, 207 authors document the history of 143 university and non-university institutes in Germany, Austria, and Switzerland, including institutions of the pharmaceutical industry and the development in former East Germany. A comprehensive name index with more than 5400 entries highlights the remarkable depth of this volume (Philippu 2004).

In addition, the volumes contain reviews, comments, and corrections related to the earlier books. For these reasons, the series should be read and understood as a whole rather than as separate individual volumes. Only by considering all volumes together does the full importance of this work become clear.

**Fig. 3:**
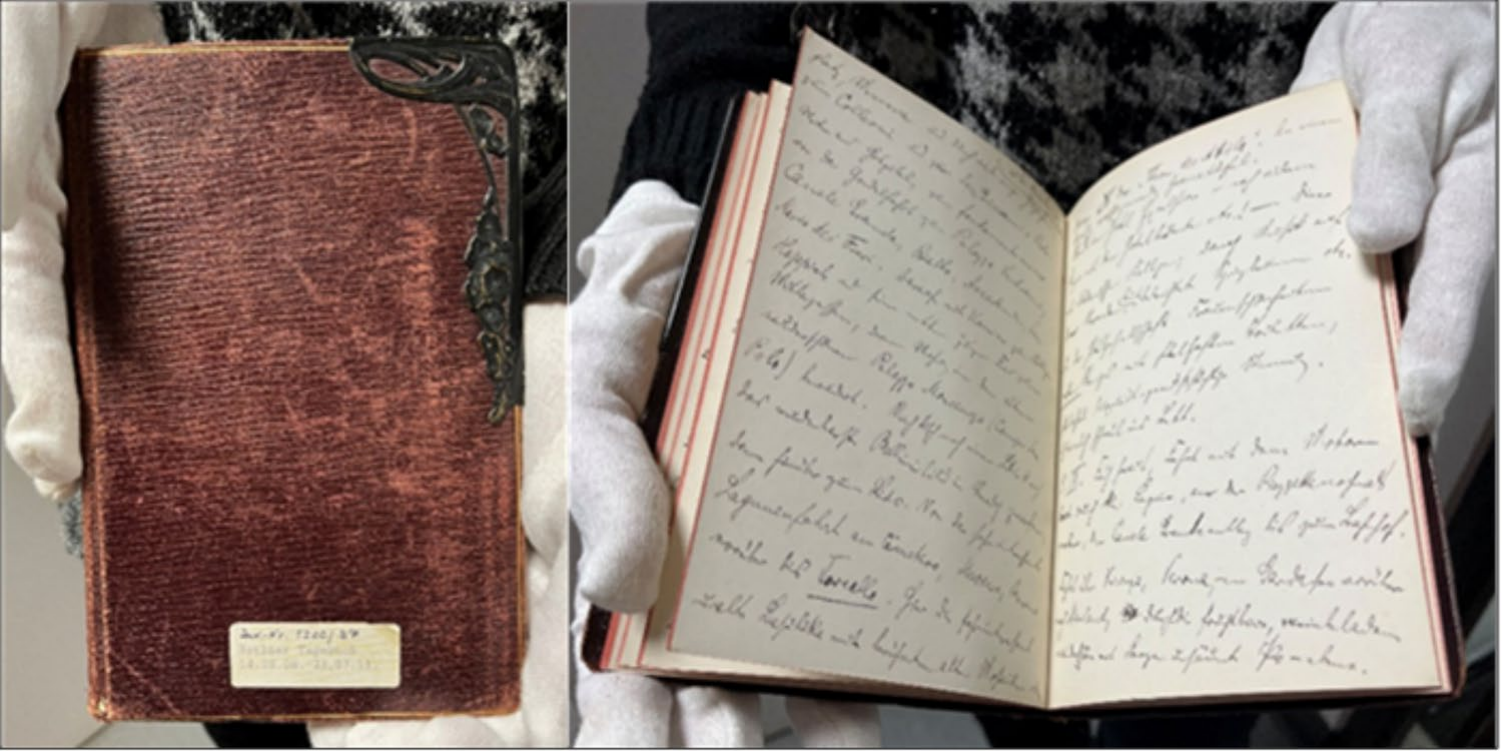
One of the 30 original notebooks of Wolfgang Heubner’s diary at the DGPT Archive (Heubner Tagebücher, ArchMHH Dep. 13, Nr. 117–121), photo by the author

Another primary source is the *Diary of Wolfgang Heubner* (*Tagebücher Wolfgang Heubner*, ArchMHH Dep. 13, Nr. 117–121), one of the key figures in German pharmacology during the first half of the twentieth century (Philippu 2017). His 30 notebooks, covering the years 1908, 1911, and 1917–1956, are written in his difficult-to-read old German handwriting (Fig. 3). Thanks to the dedicated efforts of Erich Muscholl, who transcribed and digitized them, these diaries are now easier to read and, consequently, more accessible. Both the original manuscripts and the transcriptions are preserved in the DGPT’s archive. A digital version can be accessed via the Pharmacological Institute in Hanover. In his diaries, Heubner recorded his personal views on contemporary events, providing insights not only into developments in the world of pharmacology but also into the historical context of the era, its politics and everyday life.

Wolfgang Heubner, whose life was closely connected to German history and the development of pharmacology in Germany, is well documented, partly thanks to his diaries. As a result, several studies have focused on his life and work. One example is Nils Kessel (2008), “Biographie als Disziplinentradition. Von der Idealisierung des Pharmakologen Wolfgang Heubner (1877–1957)” (Kessel 2008), which looked at Heubner’s role during and after the National Socialist period. Kessel shows that after the war, Heubner was presented as a model pharmacology professor, known for continuity, honesty, and scientific objectivity. This also helped younger professors to distance themselves from their own actions during that time, while his story made it easier to explain and justify the discipline to future generations.

### Sources on the DGPT and the involvement of pharmacology during the Nazi era

Regarding the founding of the German Pharmacological Society (DPhG), the precursor to the DGPT, in 1920, the most significant source is the work of Erich Muscholl, who established and curated the society’s archive after his retirement. Through extensive correspondence with relatives, students, and colleagues of early pharmacologists, he compiled a unique historical collection. His summary article on the society’s foundation and its first 25 years “Gründungsgeschichte und die ersten 25 Jahre der Deutschen Pharmakologischen Gesellschaft” (Muscholl 1995) remains a central reference for this period and was published in the DGPT Newsletter (DGPT Mitteilungen).

The Second World War and the Nazi era have been interpreted from various historical perspectives, which are examined in a wide range of publications.

**Fig. 4:**
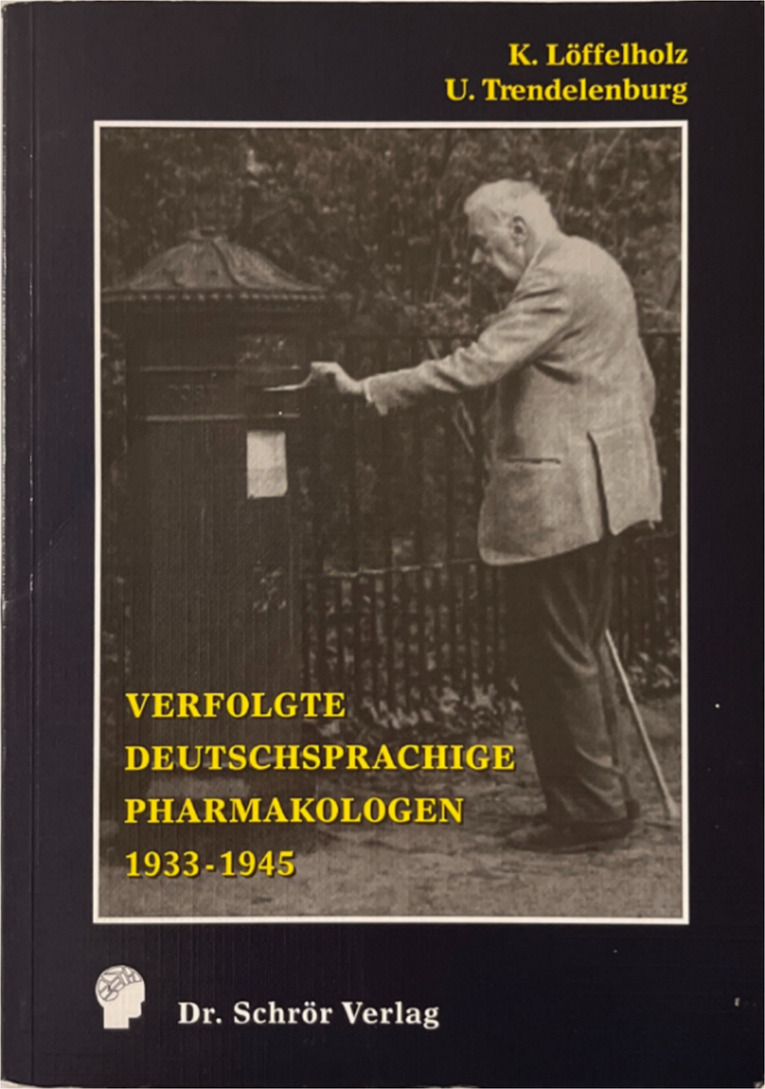
Cover of *Verfolgte deutschsprachige Pharmakologen 1933–1945*, second edition (Löffelholz and Trendelenburg 2008)

Löffelholz and Trendelenburg have examined the persecution of pharmacologists under the Nazi regime, documenting the biographies of affected individuals and offering insights into the scientific and political context in *Verfolgte deutschsprachige Pharmakologen 1933–1945* (Löffelholz and Trendelenburg 2008). This work also addresses the emigration of persecuted scholars, their countries of exile, and early post-war restitution efforts. The first edition (Trendelenburg 2006) appeared in 2006, followed 2 years later by a revised and more thorough second edition, expanded by Löffelholz (Löffelholz and Trendelenburg 2008; Fig. 4).

Expanding upon this biographical foundation, Mispagel and Seifert (2024) analyzed the scientific output and bibliometric impact of 71 persecuted pharmacologists in their paper “Scientific, bibliometric and biographical analysis of 71 Jewish and dissident pharmacologists persecuted in Germany between 1933 and 1945” (Mispagel and Seifert 2024), focusing on their publications in Naunyn–Schmiedeberg’s Archives of Pharmacology. Their findings underscore the devastating intellectual loss for German pharmacology due to persecution and expulsion, while showing how countries such as the USA and Great Britain benefited from the influx of these scholars.

In addition, in a second paper: “Biographical analysis of 32 pharmacologists persecuted under the Nazi regime: scientific careers between persecution, emigration, and new beginnings” (Mispagel and Seifert 2025), the authors look at how the careers of 32 German-speaking pharmacologists changed when they were forced to leave Germany during the Nazi time. Drawing upon the biographies by Löffelholz and Trendelenburg (2008), the study further examines the pharmacologists’ publications in key journals. The results show that younger scientists often managed to build strong careers in their new countries, while older professors usually had more difficulties. The study also gives detailed examples of ten pharmacologists who continued their work abroad thanks to their strong commitment and international support. It reminds us how political oppression can harm science and why freedom in science must be protected.

Further contextualization is provided by Schneider et al. in their “Historical analysis of the directors of university institutes of pharmacology in Germany and Austria in the period 1918–1963” (Schneider et al. 2025), which examines the directors of pharmacological institutes in Germany and Austria and their political affiliations. Their comprehensive study demonstrates how deeply pharmacology became entangled with the structures of National Socialism, while also revealing the continuities that persisted within the academic system after the war.

Building on this, Goebels et al. analyzed the role of the DGPT during and after the Nazi era, focusing on its institutional behavior and post-war reconstruction. The paper “The German Pharmacological Society during the Nazi regime (1933–1945) and its resurrection after World War II” (Goebels et al. 2025) shows that while Jewish members were never formally excluded, the society tolerated Nazi sympathizers and failed to critically address its past until an official apology was issued in 2014. The study also identifies 23 additional persecuted members for future analysis.

After 1945, the division of Germany into two states led to separate developments within pharmacology. Fritz Markwardt documented this period mainly from the East German perspective in an article published in the DGPT Newsletter. His article “Zur Entwicklung der Gesellschaft für Pharmakologie und Toxikologie der DDR” (Markwardt 1995) describes the development and organizational structure of the pharmacological landscape in the GDR and the collaboration between East and West German pharmacologists.

A second article, “Die Vereinigung der Gesellschaft für Pharmakologie und Toxikologie der DDR (GPT-DDR, nach dem 02.10. 1990 GPT) mit der Deutschen Gesellschaft für Pharmakologie und Toxikologie (DGPT) 1990/91” (Klinger and Müller 1995), published in the same issue of the DGPT Newsletter describes the merging of the two societies following German reunification and the difficulties that came with it. The unification of the societies laid the foundation for the further development of the DGPT into its current form.

**Fig. 5:**
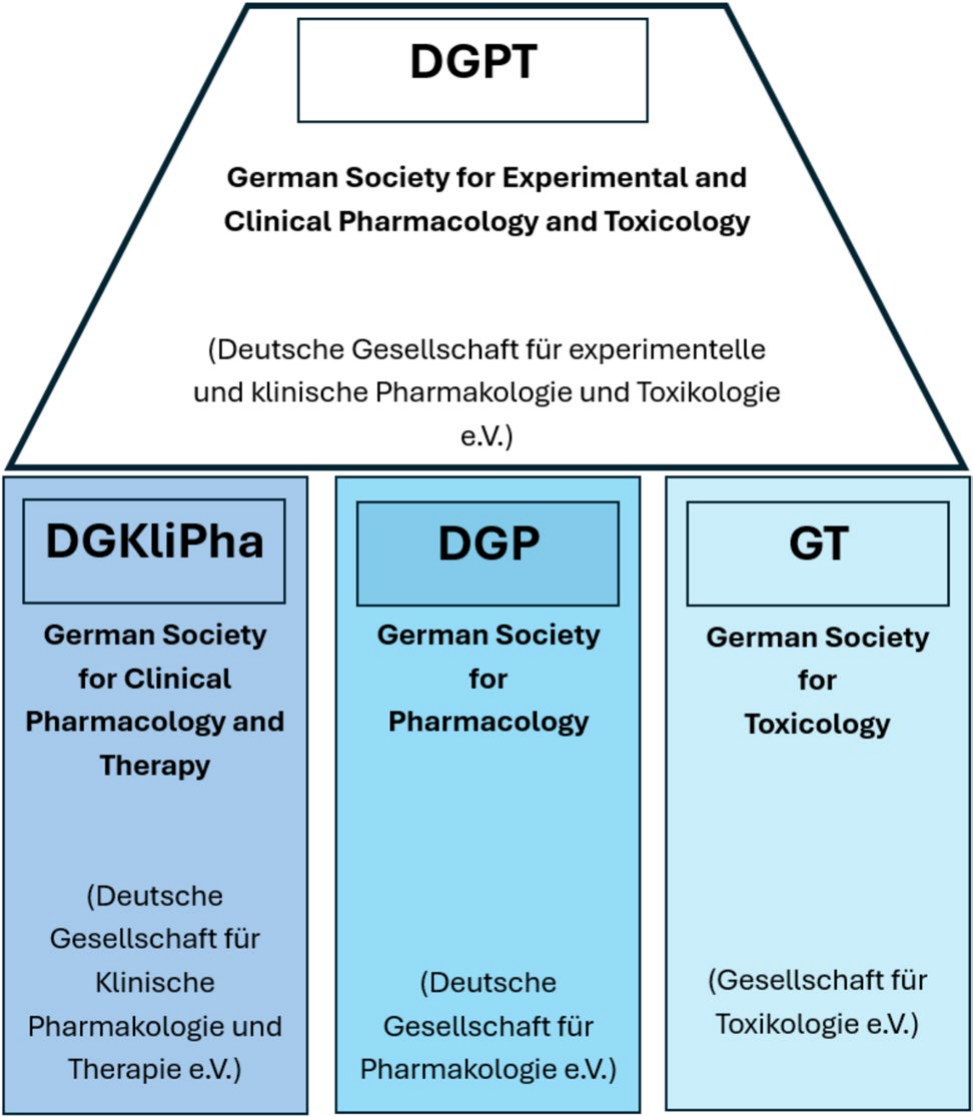
Current organizational chart of the DGPT and its branches (Über die DGPT, https://dgpt-online.de/ueber-die-dgpt/, accessed 5 Dec 2025; English translations from Organisational remarks, https://gpts-kongress.de/general-information/organisational-remarks, accessed 5 Dec 2025)

To mark its 100th anniversary in 2020, the DGPT published a booklet: *Die Deutsche Gesellschaft für Experimentelle und Klinische Pharmakologie und Toxikologie e.V. (DGPT)* offering an overview of its current structure. The DGPT serves as the umbrella organization and oversees the following equal branches: the Deutsche Gesellschaft für Klinische Pharmakologie und Therapie e.V. (DGKliPha), the Deutsche Gesellschaft für Pharmakologie e.V. (DGP), and the Deutsche Gesellschaft für Toxikologie e.V. (GT). The booklet offers a picture of the society’s present state and organizational setup and is available directly from the society. In addition, the websites of both the DGPT as the umbrella organization and its branches serve as valuable sources of information. Figure 5 illustrates the current organizational chart of the DGPT for reference (Über die DGPT. https://dgpt-online.de/ueber-die-dgpt/. Accessed 5 Dec 2025; Fig. 5).

Another aspect of the DGPT that was studied is its awards. Two studies looked at two of these awards and are worth mentioning.

Halling et al., in “The Gender Award Gap in German Medical Societies 2000–2023: the Fritz-Külz-Award as an Example,” analyze 1213 awards granted by 183 German medical societies between 2000 and 2023 (Halling et al. 2025). Using the Fritz-Külz Award of the DGPT as a representative case, the study provides a comprehensive overview of award practices in Germany’s medical societies. The results show that although the Gender Award Gap has steadily narrowed, gender imbalances persist, especially for honorary distinctions and more highly endowed research prizes. The authors call for greater transparency in nomination and application procedures within the medical societies to help identify and reduce structural disadvantages.

In a second study, Steinert and Seifert, in “The Schmiedeberg Medal of the German Society for Experimental and Clinical Pharmacology and Toxicology: a biographical and bibliometric analysis of the 47 recipients from 1956 to 2024,” conducted the first comprehensive biographical and bibliometric study of the DGPT’s highest scientific award, the Schmiedeberg Medal (Steinert and Seifert 2025). The study systematically analyzed 47 recipients of the Schmiedeberg Medal, including career trajectories, publication records, and academic backgrounds. Using bibliometric data, the authors examined trends in publication productivity, collaboration, journal choice, and research focus over time. The study provides a structured framework for understanding the careers and scientific impact of the medal recipients in pharmacology.

### The* Naunyn–Schmiedeberg’s Archives of Pharmacology *and related articles on bibliometric analysis

**Fig. 6:**
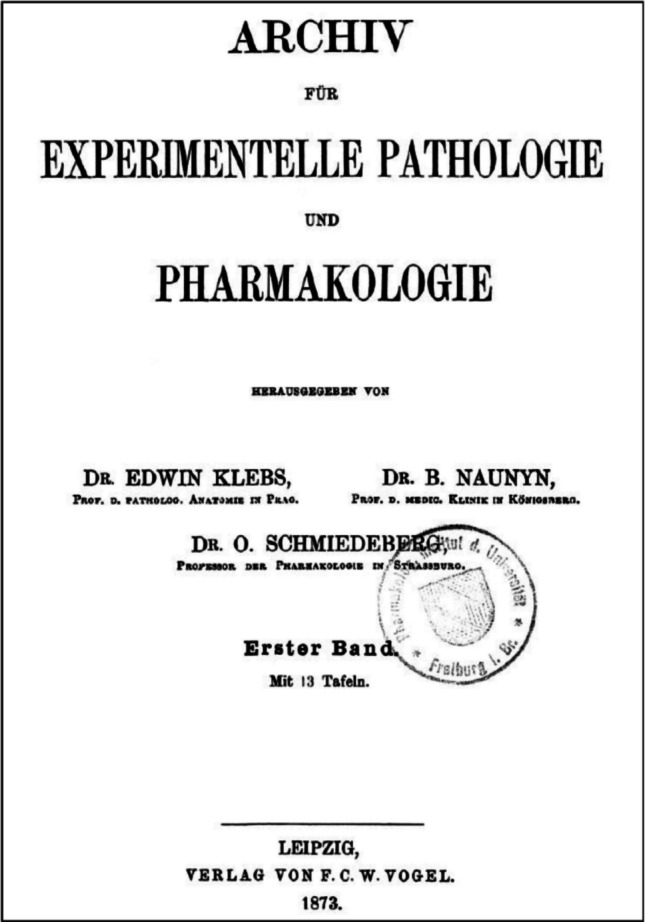
Cover of the first volume of *Naunyn–Schmiedeberg’s Archives of Pharmacology*, then called *Archiv für Experimentelle Pathologie und Pharmakologie* (*Archiv für experimentelle Pathologie und Pharmakologie* 1.1873)

The history of German pharmacology is inseparable from that of its official journal the *Naunyn–Schmiedeberg’s Archives of Pharmacology* (*NSAP*), founded in 1873, over 25 years before the establishment of the German Pharmacological Society (Fig. 6). For its 125th anniversary in 1998, Klaus Starke published a detailed study tracing the journal’s origins, editorial evolution, major publications (1873–1972), and its close connection to the DGP/DGPT. His paper “A History of *Naunyn–Schmiedeberg’s Archives of Pharmacology*” (Starke 1998) illustrates how the journal’s development mirrors the trajectory of pharmacology itself and provides bibliometric insights into the society’s scientific impact. The article further illustrates how, during the 1970 s, the *NSAP* transitioned from German to English as its mandatory language, a requirement that remains in place to this day and explains why articles, like this one on German pharmacology, are published in English. In addition, the switch in the mandatory language in the *NSAP* is discussed in Dats et al. 2023, Basol and Seifert 2024a, b, and Steinert and Seifert 2025; these studies are also presented in this paper.

In addition to Starke’s overview, *Naunyn–Schmiedeberg’s Archives of Pharmacology* itself serves as an important primary source. The journal not only contains medical-historical articles and official documents, such as conference opening speeches, but its scientific papers themselves are also valuable historical sources. These articles allow us to trace how pharmacological research developed over time, which topics were prioritized, and how methods and ideas changed. They can be used for meta-analyses and other studies that examine the evolution of the field. Together, all these materials provide direct insight into both the DGPT and the history of pharmacological research. All volumes of *NSAP* are accessible online through link.Springer.com ((2025) *Naunyn–Schmiedeberg’s Archives of Pharmacology*. In: SpringerLink. https://link.springer.com/journal/210/volumes-and-issues. Accessed 28 Oct 2025).

To date, several studies have examined publication behavior and the structures within the *NSAP*:

In “Gender-specific analysis of the authors and the editorial board of *Naunyn-Schmiedeberg’s Archives of Pharmacology* from 2000 to 2020” (Zehetbauer et al. 2022), Zehetbauer et al. performed a gender-specific analysis of authors and the editorial board of *Naunyn-Schmiedeberg’s Archives of Pharmacology* from 2000 to 2020. Using journal metadata, the analysis tracked the proportion of male and female contributors over time, examining trends in authorship, editorial representation, and leadership roles. The results show a gradual increase in female authorship and growing, though still limited, female representation on the editorial board, reflecting slow progress toward gender balance in the journal.

Motivated by the 150th anniversary of *Naunyn-Schmiedeberg’s Archives of Pharmacology*, Dats et al. studied how the journal has developed over time. In their study, “Bibliometric development of *Naunyn-Schmiedeberg’s Archives of Pharmacology*” (Dats et al. 2023), they used data from Editorial Reports, Clarivate, and Springer Nature. They looked at performance measures such as the impact factor and social impact, analyzed publication data, and created an algorithm to find the authors’ cities and countries. The study showed changes in where articles came from, the research topics, and the language used. It also explained how historical, cultural, and global factors shaped the journal and helped it become more international.

The bibliometric study “Bibliometric analysis of *Naunyn-Schmiedeberg’s Archives of Pharmacology* (1947-1974)” (Basol and Seifert 2024a) analyzed *Naunyn-Schmiedeberg’s Archives of Pharmacology* from 1947 to 1974, using Python and Beautiful Soup to extract publication data from SpringerLink. It mapped trends in language, authorship, and scientific focus, tracking the journal’s shift from German-language, nationally centered articles to an increasingly international, English-language orientation. The analysis also examined thematic patterns in research on cholinergic, adrenergic, and dopaminergic systems.

In a second study, Basol and Seifert analyzed 383 publications from Berlin in *Naunyn-Schmiedeberg’s Archives of Pharmacology* (1947–1974) to examine the impact of the Cold War on pharmacological research. Their 2024 study “The Cold War in pharmacology: a bibliometric analysis of Berlin’s contributions to *Naunyn‑Schmiedeberg’s Archives of Pharmacology* (1947-1974)” analyzed publication patterns, citations, language use, and research topics that were compared between West and East Berlin (Basol and Seifert 2024b). The study shows that West Berlin, supported by academic freedom, funding, and internationalization, published more papers, received more citations, and adopted English rapidly, while East Berlin’s output was limited by political repression, financial constraints, and restricted global engagement.

The 2025 study “A bibliometric study of the most-cited research articles and reviews in *Naunyn-Schmiedeberg’s Archives of Pharmacology* (1969-2024)” (Seifert and Hassan 2025) analyzed the top 100 publications, examining trends, authors, institutions, and research topics.The study highlights the journal’s evolution over time and its adaptation to changing research fields, including diversification through collections and calls for papers. It also notes recent trends in reviews and editorial measures to maintain *NSAP* as a reliable source for future research.

In addition to studies focusing on bibliometric analyses within the *NSAP*, there are also studies that include other journals and publishing avenues. One example is the study by Fox and Seifert, “Arbitrariness of bibliometric parameters: a case study on leading scientists in the German Society for Experimental and Clinical Pharmacology and Toxicology (DGPT)” (Fox and Seifert 2024). The authors analyzed members in Research.com’s 2022 ranking of top scientists, highlighting the underrepresentation of women in German pharmacology. Pharmacologists require more publications than biologists or biochemists to achieve a similar h-index. Age-dependent publication peaks and seven distinct publication patterns were identified, with pharmacology and toxicology showing similar bibliometric profiles. The study emphasizes that bibliometric rankings are arbitrary and should be complemented by assessments of genuine scientific and societal impact.

Additionally, Zöllner and Seifert analyzed 1326 articles in the non-peer-reviewed German magazine *Biospektrum* (1999–2021) in “How do German pharmacologists publish in the non-peer-reviewed science magazine *Biospektrum*?,” focusing on pharmacologists (Zöllner and Seifert 2024). Among 3197 authors, pharmacology was underrepresented, with most papers from three German institutions. Authors with doctoral degrees dominated, and women especially senior authors were underrepresented. The COVID-19 pandemic further reduced publications by female first authors. Compared to peer-reviewed journals, female pharmacologists invested less in “social impact” through *Biospektrum*, potentially limiting visibility and career advancement.

Within the *NSAP*, there are now two new online sections covering news and medical history research, which are regularly updated and provide information on new papers and developments:

1. DGPT news. In: SpringerLink. https://link.springer.com/collections/digciccahh. Accessed 8 Dec 2025
2. History of Pharmacology. In: SpringerLink. https://link.springer.com/collections/eadiahagff. Accessed 8 Dec 2025

### DGPT Archive

After his retirement, Erich Muscholl played a leading role in organizing and cataloguing the materials of the archive. Originally based in Mainz, after an interim period in Stuttgart, the archive was ultimately transferred to Hanover in October 2022, and has since been integrated into the University Archive of Hannover Medical School (MHH) at the Institute of Ethics, History, and Philosophy of Medicine. A detailed inventory of the collection is being implemented and is available on the archival information system of Lower Saxony and Bremen: Arcinsys.com (Arcinsys Navigator. https://www.arcinsys.niedersachsen.de/arcinsys/start.action?oldNodeid =. Accessed 28 Oct 2025). The materials are currently housed in the MHH Archive, Department 13. Of particular interest for historical research are the society’s minutes from the post-war period from 1947 to 1997 (Protokolle der Vorstandssitzungen, ArchMHH Dep. 13, Nr. 2–9), as well as records and minutes of the GDR Pharmacology board meetings (DDR-Pharmakologie und Pharmakologische Gesellschaft der DDR (GPT-DDR), ArchMHH, Dep. 13, Nr.19–26). The archive also includes folders explicitly dedicated to the founding documents and the historic documents of the DPhG and DGPT (ArchMHH Dep. 13, Nr. 40–43). The original founding documents are still held at the Berlin State Archives (Landesarchiv Berlin) and can be requested via their website and reference “Vereinsregisterakte von 1921; Landesarchiv Berlin, Signatur B Rep. 042—Amtsgericht Charlottenburg Nr. 26,558” (Landesarchiv Berlin – Das zentrale Staatsarchiv der deutschen Hauptstadt, https://landesarchiv-berlin.de/, accessed 9 Feb 2026).

**Fig. 7:**
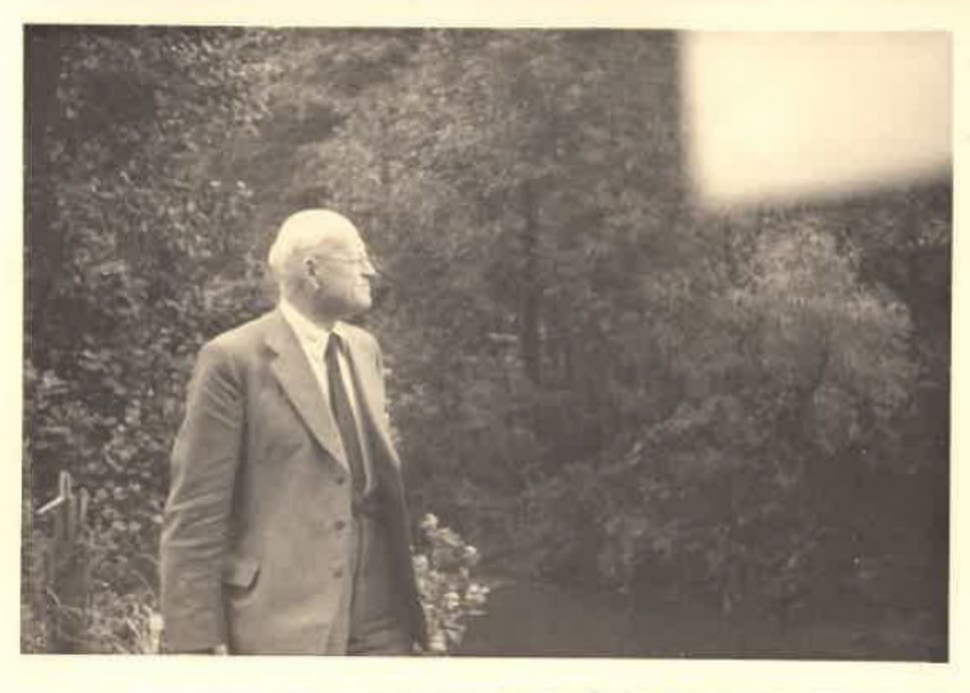
One of many historical, previously unpublished images from the DGPT archive, here showing Wolfgang Heubner at the “Pharmakologentagung” in Düsseldorf in 1948 (Foto Wolfgang Heubner, ArchMHH, Dep. 13, Nr 42). Despite thorough research, the photographer of the image could not be identified; any information in this regard is welcome. The photograph was made available to the DGPT in 1990 by H.G. Kroneberg, out of the estate of H. Weese (correspondence Kroneberg/Scholz 23.01.90, correspondence Klupp/Wolf 06.02.90, ArchMHH Dep. 13, Nr. 42)

The DGPT Archive also contains numerous previously unpublished images and documents that offer highly personal insights into the social interactions and communication of the time. In addition to photographs, the collection includes private letters, telegrams, postcards, and expense reports, as well as internal and sometimes critical discussions on pharmacological matters and personnel issues (Fig. 7).

**Fig. 8:**
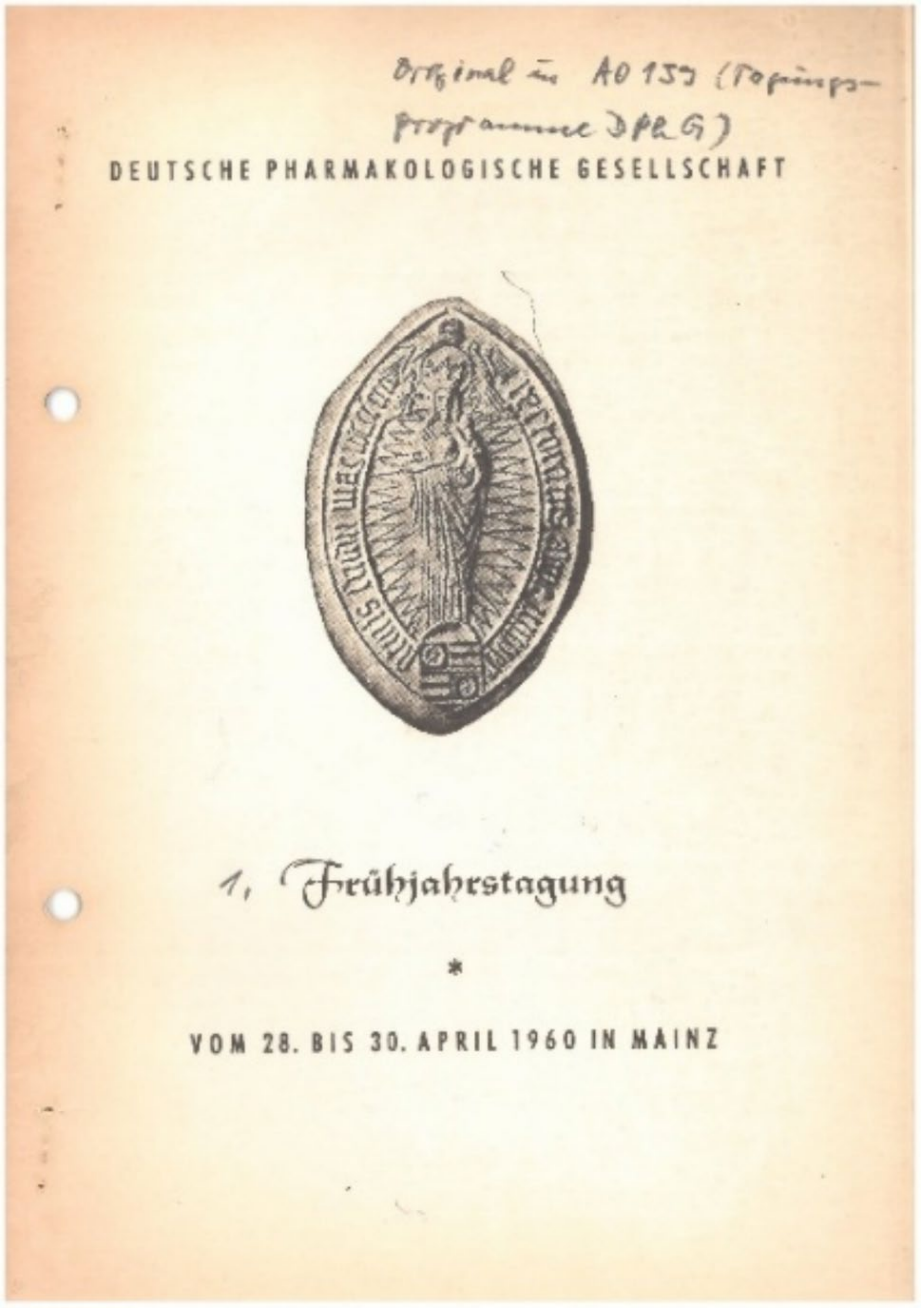
Cover of the first “Mainzer Frühjahrstagung” (Spring Congress in Mainz) (Tagungsprogramm der 1. Frühjahrstagung, ArchMHH Dep. 13, Nr. 88)

Another source for pharmacological research in the archive is the society’s collection of congress programs, including those of the Mainz Spring Congresses (Mainzer Frühjahrstagungen) held from 1960 to 2010. Later spring congresses took place in other locations and continued as annual meetings. All original programs of both the spring and annual congresses are preserved in the archive (Fig. 8).

The archive also holds printed issues of the DGPT Newsletters (DGPT Mitteilungen) from 1988 to 2001 (renamed “Forum” from 1997) (DGPT-Mitteilungen/Forum, ArchMHH Dep. 13, Nr. 103 + Nr. 104), which contain significant publications and curated information on internal association matters. In addition to the DGPT Newsletters (DGPT Mitteilungen), the DGPT News (DGPT Nachrichten) are available online via *BioSpektrum* since 2020 (BIOspektrum, https://www.biospektrum.de/suche?search_api_fulltext_op=and&s=DGPT+Nachrichten, accessed 23 Dec 2025).

Since the archive has been transferred to Hannover in 2022, several notable studies (as described above) have emerged from the Pharmacological Institute Hannover, making effective use of its resources. These works have re-examined the DGPT’s history, particularly the period of the Nazi regime, from a contemporary perspective. This research has also already had consequences: Schneider et al. revealed that Fritz Külz (1887–1949, former director of the Pharmacological Institute in Frankfurt) had been a member of a Nazi organization, a fact previously unknown (Schneider et al. 2025). As a result, the DGPT’s Fritz Külz Prize was renamed in 2025 and is now simply called the DGPT Junior Scientist Award (Nachwuchspreis der DGPT).

## Overview of the relevant sources (Table 1)

**Table 1:** Tabulated overview of the sources discussed, in alphabetical order

Author	Name	Content	Language	Source
Basol and Seifert 2024a	“Bibliometric analysis of *Naunyn–Schmiedeberg’s Archives of Pharmacology* (1947–1974)”	This bibliometric analysis of *Naunyn–Schmiedeberg’s Archives of Pharmacology* (1947–1974) highlights its shift from a national, German-language focus to internationalization and English-language adoption, boosting global visibility and citations. The study emphasizes key research trends, including cholinergic, adrenergic, and dopaminergic systems, during this transformative period	English	*Naunyn Schmiedebergs Arch Pharmacol* 397:7141–7168. 10.1007/s00210-024–03078-8
Basol and Seifert 2024b	“The Cold War in pharmacology: a bibliometric analysis of Berlin’s contributions to *Naunyn–Schmiedeberg’s Archives of Pharmacology* (1947–1974)”	A bibliometric analysis of *Naunyn–Schmiedeberg’s Archives* (1947–1974) shows that Cold War divisions strongly shaped pharmacological research in Berlin. West-Berlin, benefiting from academic freedom, funding, and international ties, published more, received higher citations, adopted English faster, and focused on different research topics than East Berlin, where political repression and limited resources hindered scientific development	English	*Naunyn Schmiedebergs Arch Pharmacol* 397:7963–7980. 10.1007/s00210-024–03115-6
Dats et al. 2023	“Bibliometric development of *Naunyn–Schmiedeberg’s Archives of Pharmacology*”	A 150-year bibliometric review of *Naunyn–Schmiedeberg’s Archives of Pharmacology* shows declining German contributions, rising international authorship, and shifts from neurotransmitter research to cancer and immunopharmacology—illustrating how scientific journals evolve with global and cultural change	English	*Naunyn Schmiedebergs Arch Pharmacol* 396:43–61. 10.1007/s00210-022–02307-2
DGPT 2020	*Die Deutsche Gesellschaft für Experimentelle und Klinische Pharmakologie und Toxikologie e.V*	100th-anniversary booklet presenting the DGPT’s structure, including its constituent branches (DGKliPha, DGP Düsseldorf, GT); provides an overview of the society’s current state and organization	German	DGPT
Fox and Seifert 2024	“Arbitrariness of bibliometric parameters: a case study on leading scientists in the German Society for Experimental and Clinical Pharmacology and Toxicology (DGPT)”	An analysis of leading German pharmacologists and toxicologists on Research.com shows that rankings shift markedly depending on the metrics used. Clear publication patterns and field differences emerge, highlighting that bibliometric indicators alone are too limited for fair hiring and funding decisions and should be balanced with broader measures of scientific and societal impact	English	*Naunyn Schmiedebergs Arch Pharmacol* 397:8925–8942. 10.1007/s00210-024–03195-4
Goebels et al. 2025	“The German Pharmacological Society during the Nazi regime (1933–1945) and its resurrection after World War II”	Analysis of DGPT during and after the Nazi era, highlighting institutional tolerance of Nazi sympathizers, delayed reckoning until 2014, and identification of 23 additional persecuted members	English	*Naunyn–Schmiedeberg’s Arch Pharmacol.* 10.1007/s00210-025–04605-x
Halling et al. 2025	“The Gender Award Gap in German medical societies 2000–2023: the Fritz-Külz-Award as an example”	Comprehensive analysis of prizes in German medical societies (2000–2023), revealing progress in gender equality but persistent imbalances in prestigious awards. It highlights the need for greater transparency in nomination and selection processes	English	*Naunyn–Schmiedeberg’s Arch Pharmacol.* 10.1007/s00210-025–03892-8
Heubner 1908–1956	*Tagebücher Wolfgang Heubner*	Handwritten diaries documenting personal views on pharmacology, contemporary events, politics, everyday life, and social conditions; 30 notebooks, transcribed and digitized by Erich Muscholl	German	Arch MHH Dep. 13, Nr 117–121
K essel 2008	“Biographie als Disziplinentradition. Von der Idealisierung des Pharmakologen Wolfgang Heubner (1877–1957)”	Paper about Wolfgang Heubners role during nazi era Germany	German	Medizin, Gesellschaft, und Geschichte: Jahrbuch des Instituts für Geschichte der Medizin der Robert Bosch Stiftung 27:133–60
Klinger and Müller 1995	“Die Vereinigung der Gesellschaft für Pharmakologie und Toxikologie der DDR (GPT-DDR, nach dem 02.10. 1990 GPT) mit der Deutschen Gesellschaft für Pharmakologie und Toxikologie (DGPT) 1990/91”	Merging of the East and West German pharmacological societies after reunification and the challenges involved	German	DGPT Mitteilungen Nr. 16, Februar 1995
L indner 1957	*Zeittafeln zur Geschichte der Pharmakologischen Institute des deutschen Sprachgebiets*	Chronological overview of German-speaking pharmacology institutes, early associations, key figures	German	Cantor Verlag, Aulendorf i. Württ
Löffelholz and Trendelenburg 2008	*Verfolgte deutschsprachige Pharmakologen 1933–1945*	Examines the persecution of German-speaking pharmacologists under the Nazi regime; documents biographies of affected scholars, their emigration, countries of exile, and early post-war restitution efforts; second, revised edition of Trendelenburg 2006	German	Dr. Schrör Verlag, Frechen
Markwardt 1995	“Zur Entwicklung der Gesellschaft für Pharmakologie und Toxikologie der DDR”	Development and organization of pharmacology in the GDR and collaboration between East and West German pharmacologists	German	DGPT Mitteilungen Nr. 16, Februar 1995
Mispagel and Seifert 2024	“Scientific, bibliometric and biographical analysis of 71 Jewish and dissident pharmacologists persecuted in Germany between 1933 and 1945”	Bibliometric analysis of 71 persecuted pharmacologists (1900–1980) via *Naunyn–Schmiedeberg’s Archives*, highlighting intellectual loss for Germany and gains for the USA and UK	English	*Naunyn–Schmiedeberg’s Arch Pharmacol.* 10.1007/s00210-024–03645-z
Mispagel and Seifert 2025	“Biographical analysis of 32 pharmacologists persecuted under the Nazi regime: scientific careers between persecution, emigration, and new beginnings”	The study shows how Nazi persecution reshaped the careers of German pharmacologists, with younger émigrés often thriving abroad while older scientists struggled	English	*Naunyn–Schmiedeberg’s Arch Pharmacol.* 10.1007/s00210-025–04231-7
Muscholl 1995	“Gründungsgeschichte und die ersten 25 Jahre der Deutschen Pharmakologischen Gesellschaft”	Key source on the founding of the German Pharmacological Society (DGP), central reference for the society’s first 25 years	German	DGPT Mitteilungen Nr. 16, Februar 1995
Philippu 2004–2021	*Geschichte und Wirken der pharmakologischen* *, * *klinisch-pharmakologischen und toxikologischen Institute im deutschsprachigen Raum*	Extremely detailed, six-volume, comprehensive overview of university and non-university pharmacological institutes; includes biographies and autobiographies of influential pharmacologists; covers history and present state of German-speaking pharmacology	English, German	Berenkamp Verlag, Wattens
Philippu and Seifert 2023a	“History of pharmacology: 1—the Department of Pharmacology of the University of Tartu (Dorpat): genealogy and biographies”	Development of pharmacology in Tartu shaped by Naunyn, Buchheim, and Schmiedeberg	English	*Naunyn–Schmiedeberg’s Arch Pharmacol* 396:5–17. 10.1007/s00210-022–02328-x
Philippu and Seifert 2023b	“History of pharmacology:2—The Institute of Pharmacology of the University of Strasbourg: genealogy and biographies”	Focus on Schmiedeberg’s Strasbourg institute (1872–1918) as a center of pharmacological training and academic influence	English	*Naunyn–Schmiedeberg’s Arch Pharmacol* 396:19–33. 10.1007/s00210-022–02336-x
Schneider et al. 2025	“Historical analysis of the directors of university institutes of pharmacology in Germany and Austria in the period 1918–1963”	Study of German and Austrian pharmacology institute directors (1918–1963) and political affiliations, showing entanglement with National Socialism and post-war continuities	English	*Naunyn–Schmiedeberg’s Arch Pharmacol.* 10.1007/s00210-025–04342-1
Seifert and Hassan 2025	“A bibliometric study of the most-cited research articles and reviews in *Naunyn–Schmiedeberg’s Archives of Pharmacology* (1969–2024)”	Analysis of the top 100 most-cited research articles and reviews in *Naunyn–Schmiedeberg’s Archives of Pharmacology* (1969–2024), revealing trends in citations, authorship, and global contributions. The results inform editorial strategies and highlight shifts in research focus over time	Englisch	*Naunyn Schmiedebergs Arch Pharmacol.* 10.1007/s00210-025–04471-7
Starke 1998	“A History of *Naunyn–Schmiedeberg’s Archives of Pharmacology*”	Detailed history of *Naunyn–Schmiedeberg’s Archives* (1873–1972), covering origins, editorial evolution, major publications, and links to DGP/DGPT; illustrates journal’s role in shaping German pharmacology and provides bibliometric insights	English	*Naunyn–Schmiedeberg’s Arch Pharmacol* 358:1–109. 10.1007/PL00005229
S teinert and Seifert 2025	“The Schmiedeberg Medal of the German Society for Experimental and Clinical Pharmacology and Toxicology: a biographical and bibliometric analysis of the 47 recipients from 1956 to 2024”	First in-depth analysis of Schmiedeberg Medal recipients, highlighting their exceptionally long, productive careers and lasting impact on pharmacology	English	*Naunyn–Schmiedeberg’s Arch Pharmacol.* 10.1007/s00210-025–04260-2
Various authors	DGPT Archive, MHH, Department 13	Includes board minutes, newsletters, founding documents, correspondence, personal papers, and other materials documenting the society’s history. inventory accessible via Arcinsys	German, English	Institute of Ethics, History, and Philosophy of Medicine, Hannover Medical School
Various authors	*Naunyn–Schmiedeberg’s Archives of Pharmacology*	Primary source containing medical-historical articles, official documents (e.g., conference speeches), and scientific papers that reflect the development of pharmacological research, methods, topics, and ideas; usable for meta-analyses and historical studies	German, English	SpringerLink. https://link.springer.com/journal/210/volumes-and-issues
Z ehetbauer et al. 2022	“Gender-specific analysis of the authors and the editorial board of *Naunyn–Schmiedeberg’s Archives of Pharmacology* from 2000 to 2020”	From 2000 to 2020, female authors in *Naunyn–Schmiedeberg’s Archives* increased until 2008 but then plateaued around 30%, with women holding only ~ 15% of senior authorships. In contrast, their representation on the editorial board rose substantially after 2016, reflecting targeted appointments	English	*Naunyn Schmiedebergs Arch Pharmacol* 395:39–50. 10.1007/s00210-021–02166-3
Zöllner and Seifert 2024	“How do German pharmacologists publish in the non-peer-reviewed science magazine *Biospektrum*?”	This study analyzes pharmacologists’ publications in the non-peer-reviewed German magazine *Biospektrum* (1999–2021), showing underrepresentation of pharmacology and limited engagement in outlets that enhance broader scientific visibility	English	*Naunyn Schmiedebergs Arch Pharmacol* 397:1889–1900. 10.1007/s00210-023–02740-x

## Historical contextualization

To understand the developments of pharmacology in Germany, it is important to view the events within their historical and political context. An early and significant event was the founding of the *Archiv für experimentelle Pathologie und Pharmakologie* (Today: *Naunyn–Schmiedeberg’s Archives of Pharmacology*) in 1873 (Starke 1998), during the time of the German Empire (Deutsches Kaiserreich) (1871–1918) (Schubert and Klein 2011). This was also the period during which Schmiedeberg (1838–1921), as the successor to Buchheim’s teachings (1820–1879), shaped the early development of pharmacology as an independent discipline. Schmiedeberg held a professorship in Strasbourg from 1872 to 1918 (Philippu and Seifert 2023a, b). In this period, there was also a predecessor of the German Pharmacological Society (DPhG): the Pharmacologists’ Association (Pharmakologenvereinigung), which existed from 1900 to 1907 (Lindner 1957). After it was dissolved, there was no pharmacological society in Germany for several years. It was only in 1920, after the First World War and during the Weimar Republic (1918–1933), that the DPhG was established (Schubert and Klein 2020). For contextualization, it was not until 1928 that Alexander Fleming discovered the antibacterial activity of penicillin (Tan and Tatsumura 2015).

The society continued to exist during the time of National Socialism from 1933 on and throughout the Second World War (1939–1945). In both World Wars, pharmacology in Germany was applied for military purposes, which intensified research efforts in certain fields. Research focused on improving military outcomes by treating wounded soldiers (Sabbatani and Fiorino 2017), developing chemical warfare agents and their antidotes (Fitzgerald 2008), enhancing troop performance with stimulants such as Pervitin (Sölle 2021) and advancing antimalarials (Brabin 2014) and early antibiotics. The fact that more soldiers died from infectious diseases than from direct combat during the wars underscores the critical importance of the latter (Shanks 2014). These developments illustrate how pharmacological research served both medical and strategic military objectives.

After the Second World War and Germany’s political division, separate pharmacological societies developed in East and West Germany. During this time, international scientific societies also grew in importance. One example is the British Pharmacological Society, which was joined by several German émigré scientists during the Second World War, like Marthe Vogt or Wilhelm Feldberg (Löffelholz and Trendelenburg 2008). In Germany, separate pharmacological societies developed in East and West Germany. Although cooperation between them existed, it was politically controlled (Markwardt 1995).

With the political reunification of Germany in 1990, the two societies were also united, as the East German society joined the one in the West. Since then, developments in this field have moved forward together again (Klinger and Müller 1995; Markwardt F 1995, Schubert and Klein 2020). From 1993 on, the pharmacological society adopted its new and current name Deutsche Gesellschaft für experimentelle und klinische Pharmakologie und Toxikologie e.V. (DGP—Pharmakologie in Deutschland, https://pharmakologie.org/dgp/, accessed 4 Feb 2026).

Up to the present day, international collaborations in pharmacology have continued to increase, with both productivity and cooperative research steadily growing (Steinert and Seifert 2025).

**Fig. 9:**
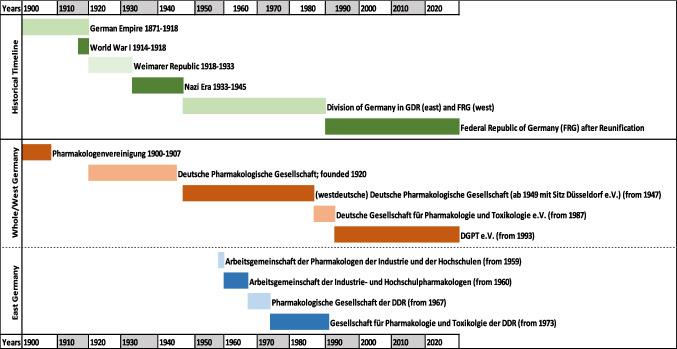
A comparative timeline of the pharmacological societies in East and West Germany and their relation to German history

Figure 9 shows the development of the DGPT in relation to Germany’s political history (Fig. 9). It clearly demonstrates that there has always been a close and mutual connection between Germany’s political and social changes and the development of its pharmacological societies.

## Further information

For additional information, the following contacts are available:

- Hochschularchiv der Medizinischen Hochschule Hannover—Building J1, Carl-Neuberg-Straße 1, 30,625 Hannover; email: hochschularchiv@mh-hannover.de.

- The DGPT Archive is located within the university archive and is open to all interested researchers after contacting the archivists via email or Arcinsys.com (https://www.arcinsys.niedersachsen.de/arcinsys/start.action?oldNodeid =).

- Continued historical research on medical and pharmacological topics is strongly encouraged, and the archive’s materials are intended to remain accessible to anyone wishing to contribute to this important work.

- Institut für Pharmakologie, Medizinische Hochschule Hannover—for questions concerning content of the DGPT Archive and access to the digital version of the Heubner Diaries, contact Prof. Roland Seifert.

- DGPT—for current information about the organization and activities of the society: office: Deutsche Gesellschaft für experimentelle und klinische Pharmakologie und Toxikologie (DGPT), Grafenberger Allee 100, 40,237 Düsseldorf; email: service@dgpt-online.de.

Overview of the most important online sources are as follows:

- Deutsche Gesellschaft für experimentelle und klinische Pharmakologie und Toxikologie e.V., https://dgpt-online.de/, accessed 5 Dec 2025.

- GT–Gesellschaft für Toxikologie–Toxikologie Toxikologen, https://toxikologie.de/, accessed 5 Dec 2025.

- Pharmakologie in Deutschland, https://pharmakologie.org/, accessed 5 Dec 2025.

- DGPT newsletter in *BIOspektrum*, https://www.biospektrum.de/suche?search_api_fulltext_op=and&s=DGPT+Nachrichten, accessed 23 Dec 2025.

- DGPT news. In: SpringerLink, https://link.springer.com/collections/digciccahh, accessed 8 Dec 2025.

- History of Pharmacology. In: SpringerLink, https://link.springer.com/collections/eadiahagff, accessed 8 Dec 2025.

## Summary and discussion

The history of the DGPT is a very good reflection of how society has developed and changed over time. It shows how pharmacology grew together with the major events of German history from the early years of the discipline, through times of war and political division, and later during reunification. The development of the DGPT followed these social and political changes very closely, showing how science is influenced by its surroundings. The historical materials also show the people behind the institutions, their ideas, challenges, and achievements over time. Altogether, the history of the DGPT helps us understand that science and society are deeply connected and that this connection continues to shape the responsibilities of the DGPT and the wider pharmacological community today.

In addition, it becomes clear that contemporary historical developments are well reflected in the *NSAP*.

By bringing together the most important historical sources, this article provides a foundation for future research. Later studies may examine specific periods, such as the Cold War years, or look at international collaborations. They can also explore how the history of the DGPT relates to larger questions about identity, memory, and social change. Learning from the past does more than preserve history; it also helps the society understand its role and responsibilities for the future. It is strongly encouraged for scientists to join the research on the topic of the history of the DGPT and pharmacology itself and to make use of the available data.

## Future perspective

Although many aspects of German pharmacology have already been studied in detail, important areas remain insufficiently explored. One such area concerns the relationships between the DGPT and its predecessor societies and other international scientific organizations. Evidence of close international cooperation can be found in publications from the Nazi and post-war periods. These include, for example, the influence of emigré pharmacologists who continued their scientific work in their countries of exile (Löffelholz and Trendelenburg 2008; Mispagel and Seifert 2024, 2025). In addition, the awarding of the Schmiedeberg Medal by the DGPT to international pharmacologists, such as Julius Axelrod, Göran Liljestrand, or Sir Henry Dale, reflects a deliberate effort to promote internationalization (Steinert and Seifert 2025). Together, these examples clearly demonstrate international scientific exchange.

However, additional research is needed to better understand the full extent of these collaborations. Future studies could focus on the numerous scientific meetings and conferences organized by the DGPT, such as the “Jahrestagungen” and the “Mainzer Frühjahrstagungen,” as well as on the participation of members from other national and international societies. Another important aspect would be the involvement of German pharmacologists in meetings and events organized by foreign societies. A prominent example is the IUPHAR World Congress of Pharmacology, which was held in Munich in 1998 (Past IUPHAR Congresses, https://iuphar.org/past-iuphar-world-congresses/, accessed 8 Feb 2026).

Other professional societies are also actively working on documenting their own medical and scientific history. For example, the British Pharmacological Society (BPS) offers a historical overview of the society on its website (Cuthbert 2006) and also provides its members with access to its archives, the contents of which can be consulted online via the Wellcome Collection (British Pharmacological Society. In: Wellcome Collection, https://wellcomecollection.org/works/pq9uae64, accessed 8 Feb 2026).

Similarly, the American Society for Pharmacology and Experimental Therapeutics (ASPET) published an overview article to mark the 100th anniversary of the society (Parascandola 2007).

Another possible way to illustrate collaborations is the creation of publication and collaboration networks, which would reflect concrete scientific projects and collaborative relationships.

Overall, the history of pharmacology should not be viewed in isolation within individual countries, but rather in the context of its integration into the network of international pharmacology. Such approaches would provide a more complete picture of international cooperation in pharmacology.

Another important topic that has so far been examined only on the basis of limited published material is the role of pharmacology in the German Democratic Republic (GDR) and its importance for pharmacology in Germany as a whole, as well as for the international scientific community. Although numerous historical documents related to this topic are available in the DGPT Archive, they have not yet been fully analyzed. This shows that there is significant potential for further and more detailed research. Currently, an article focusing on publication practices in the GDR is already in preparation. This work could represent an important first step toward a more systematic and comprehensive historical evaluation of pharmacology in the GDR.

Overall, the history of the DGP/DGPT, while partly well documented and discussed in this article, is still far from being fully investigated from a historical perspective. We hope that this study encourages other researchers to further explore this interesting and scientifically important history.

## Data Availability

All source data for this work (or generated in this study) are available upon reasonable request.

## Abbreviations

DDR: Deutsche Demokratische Republik
DGKliPha: Deutsche Gesellschaft für Klinische Pharmakologie und Therapie e.V.
DGP: Deutsche Gesellschaft für Pharmakologie e.V.
DGPT: Deutsche Gesellschaft für experimentelle und klinische Pharmakologie und Toxikologie e.V.
DPhG: Deutsche Pharmakologische Gesellschaft
Fig.: Figure
FRG: Federal Republic of Germany
GDR: German Democratic Republic
GT: Deutsche Gesellschaft für Toxikologie e.V.
MHH: Medizinische Hochschule Hannover (Hannover Medical School)
Nr.: Number
*NSAP*: *Naunyn-Schmiedeberg’s Archives of Pharmacology*
p.: Page
Tbl.: Table
